# Carbon­yl(*N*-nitroso-*N*-oxido-1-naphtylamine-κ^2^ *O*,*O*′)(triphenyl­phosphine-κ*P*)rhodium(I) acetone solvate

**DOI:** 10.1107/S1600536809047321

**Published:** 2009-11-14

**Authors:** Johan A. Venter, W. Purcell, H. G. Visser, T. J. Muller

**Affiliations:** a Department of Chemistry, University of the Free State, PO Box 339, Bloemfontein 9300, South Africa

## Abstract

The title compound, [Rh(C_10_H_7_N_2_O_2_)(C_18_H_15_P)(CO)]·(CH_3_)_2_CO, is the second structural report of a metal complex formed with the *O*,*O*′-C_10_H_7_N_2_O_2_ (neocupferrate) ligand. In the crystal structure, the metal centre is surrounded by one carbonyl ligand, one triphenyl­phosphine ligand and the bidentate neocupferrate ligand, forming a distorted square-planar RhCO_2_P coordination set which is best illustrated by the small O—Rh—O bite angle of 77.74 (10)°. There are no classical hydrogen-bond inter­actions observed for this complex.

## Related literature

For synthesis of similar Rh complexes and information on oxidative addition products, see: Basson *et al.* (1984 ▶, 1986 ▶); Steyn *et al.* (1992 ▶); Smit *et al.* (1994 ▶); Roodt & Steyn (2000 ▶). For another structural report of a complex with the bidentate neocupferrate ligand, see: Tamaki & Okabe (1998 ▶).
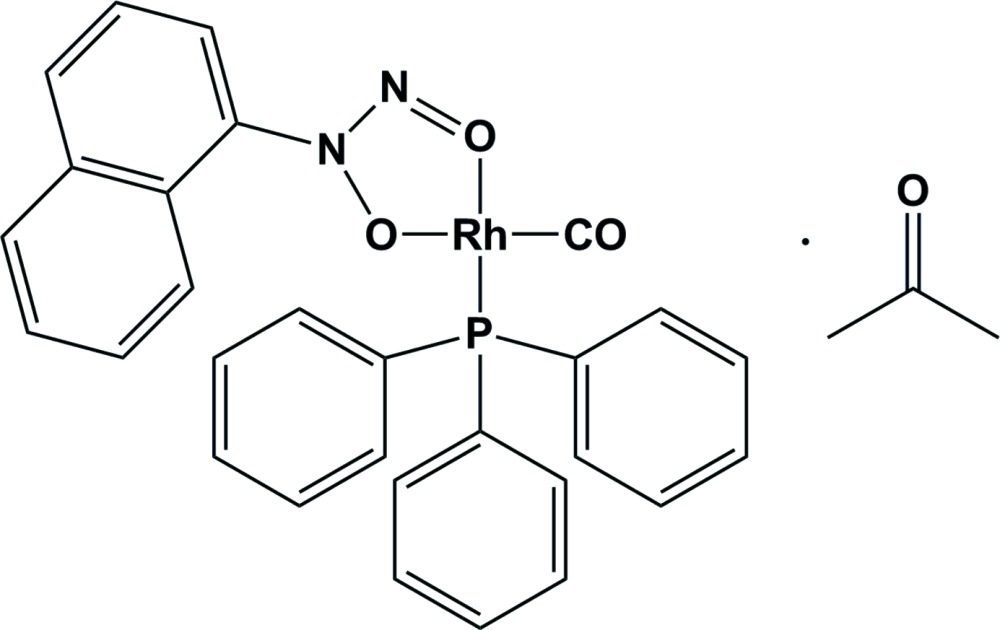


## Experimental

### 

#### Crystal data

[Rh(C_10_H_7_N_2_O_2_)(C_18_H_15_P)(CO)]·C_3_H_6_O
*M* *_r_* = 638.44
Triclinic, 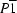

*a* = 9.709 (5) Å
*b* = 10.186 (5) Å
*c* = 15.393 (5) Å
α = 77.499 (5)°
β = 85.045 (5)°
γ = 70.279 (5)°
*V* = 1398.9 (11) Å^3^
*Z* = 2
Mo *K*α radiation
μ = 0.71 mm^−1^
*T* = 100 K
0.21 × 0.21 × 0.08 mm

#### Data collection

Bruker X8 APEXII 4K Kappa CCD diffractometer
Absorption correction: multi-scan *SADABS* (Bruker, 2004 ▶) *T*_min_ = 0.763, *T*_max_ = 0.847
23989 measured reflections
6710 independent reflections
5377 reflections with *I* > 2σ(*I*)
*R*_int_ = 0.053

#### Refinement

*R*[*F* ^2^ > 2σ(*F* ^2^)] = 0.047
*wR*(*F* ^2^) = 0.157
*S* = 1.16
6710 reflections
363 parameters
H-atom parameters constrained
Δρ_max_ = 1.75 e Å^−3^
Δρ_min_ = −1.18 e Å^−3^

### 

Data collection: *APEX2* (Bruker, 2005 ▶); cell refinement: *SAINT-Plus* (Bruker, 2004 ▶); data reduction: *SAINT-Plus* and *XPREP* (Bruker, 2004 ▶); program(s) used to solve structure: *SIR97* (Altomare *et al.*, 1999 ▶); program(s) used to refine structure: *SHELXL97* (Sheldrick, 2008 ▶); molecular graphics: *DIAMOND* (Brandenburg & Putz, 2005 ▶); software used to prepare material for publication: *WinGX* (Farrugia, 1999 ▶).

## Supplementary Material

Crystal structure: contains datablocks global, I. DOI: 10.1107/S1600536809047321/wm2279sup1.cif

Structure factors: contains datablocks I. DOI: 10.1107/S1600536809047321/wm2279Isup2.hkl

Additional supplementary materials:  crystallographic information; 3D view; checkCIF report

## Figures and Tables

**Table:** 

C1—Rh1	1.817 (4)
O2—Rh1	2.026 (3)
O3—Rh1	2.082 (2)
P1—Rh1	2.2240 (11)

**Table:** 

C1—Rh1—O2	176.15 (13)
C1—Rh1—O3	101.74 (14)
O2—Rh1—O3	77.74 (10)
C1—Rh1—P1	90.54 (12)
O2—Rh1—P1	89.92 (8)
O3—Rh1—P1	167.66 (8)

**Fig. 1.**
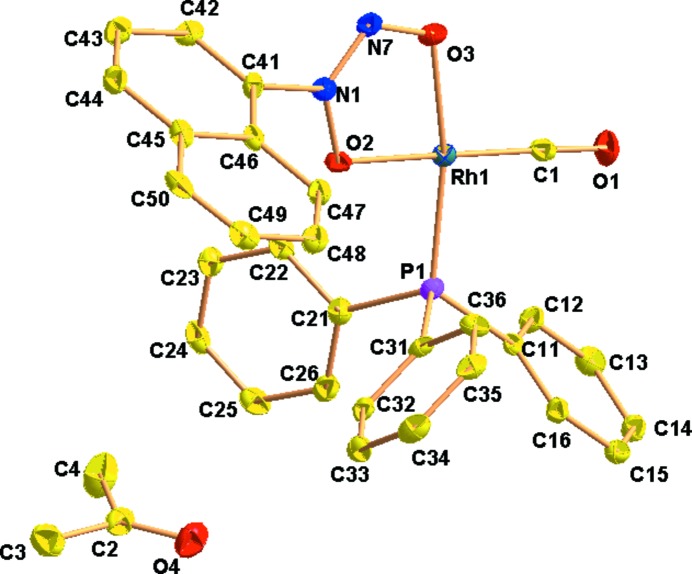
View of the complex molecule of the title compound and of the solvent molecule. Displacement ellipsoids are drawn at the 50% probability level. Hydrogen atoms are omitted for clarity.

## supplementary crystallographic information

### Comment

The title compound (Figure 1) forms part of a series of rhodium complexes used in the kinetic studies of oxidative addition reactions (Basson *et al.*, 1984, 1986; Steyn *et al.*, 1992; Smit *et al.*, 1994; Roodt & Steyn, 2000).

In the crystal structure, the Rh(I) metal centre is coordinated to one carbonyl ligand, one triphenylphosphine ligand and the bidentate neocupferrate ligand, (C_10_H_7_N_2_O_2_) to form a distorted square planar complex best illustrated by the small O–Rh–O bite angle of 77.74 (10) °. The Rh–O2 bond length of 2.026 (3) Å is significantly smaller than the Rh–O3 bond length of 2.082 (2) Å and is indicative of the larger *trans*-influence of the PPh_3_ ligand as opposed to the carbonyl ligand. This is the second structural report involving the neocupferrate ligand (Tamaki & Okabe, 1998). There is no classical hydrogen interaction observed for this complex.

### Experimental

A solution of [Rh_2_Cl_2_(CO)_4_] was prepared by refluxing a solution of hydrated RhCl_3_ in DMF for approximately 30 minutes. An equivalent amount of *N*-hydroxy-*N*-nitrosonaphtylamine (neocupf) was added to this solution to produce [Rh(neocupf)(CO)(PPh_3_)], which was isolated through precipitation with water. The title compound was obtained by leaving a 5 cm^3^ beaker containing a concentrated acetone solution of [Rh(neocupf)(CO)(PPh_3_)] uncovered at room temperature. Well shaped yellow crystals formed within 4 h.

### Refinement

The methylene, aromatic and methyl H atoms were placed in geometrically idealized positions (C—H = 0.93 – 0.98 Å) and constrained to ride on their parent atoms with *U*_iso_(H) = 1.2*U*_eq_(C) for methylene and aromatic protons and *U*_iso_(H) = 1.5*U*_eq_(C) for methyl protons, respectively. The highest residual electron density was located 0.99 Å from H4A and the deepest hole was 0.85 Å from Rh1.

### Crystal data

**Table:**

[Rh(C_10_H_7_N_2_O_2_)(C_18_H_15_P)(CO)]·C_3_H_6_O	*Z* = 2
*M**_r_* = 638.44	*F*(000) = 652
Triclinic, *P*1	*D*_x_ = 1.516 Mg m^−^^3^
Hall symbol: -P 1	Mo *K*α radiation, λ = 0.71069 Å
*a* = 9.709 (5) Å	Cell parameters from 5578 reflections
*b* = 10.186 (5) Å	θ = 2.1–28.1°
*c* = 15.393 (5) Å	µ = 0.71 mm^−^^1^
α = 77.499 (5)°	*T* = 100 K
β = 85.045 (5)°	Plate, yellow
γ = 70.279 (5)°	0.21 × 0.21 × 0.08 mm
*V* = 1398.9 (11) Å^3^	

### Data collection

**Table:**

Bruker X8 APEXII 4K Kappa CCD diffractometer	6710 independent reflections
Radiation source: sealed tube	5377 reflections with *I* > 2σ(*I*)
graphite	*R*_int_ = 0.053
φ and ω scans	θ_max_ = 28°, θ_min_ = 1.4°
Absorption correction: multi-scan *SADABS* (Bruker, 2004)	*h* = −11→12
*T*_min_ = 0.763, *T*_max_ = 0.847	*k* = −13→13
23989 measured reflections	*l* = −19→20

### Refinement

**Table:**

Refinement on *F*^2^	0 restraints
Least-squares matrix: full	H-atom parameters constrained
*R*[*F*^2^ > 2σ(*F*^2^)] = 0.047	*w* = 1/[σ^2^(*F*_o_^2^) + (0.0853*P*)^2^ + 0.0168*P*] where *P* = (*F*_o_^2^ + 2*F*_c_^2^)/3
*wR*(*F*^2^) = 0.157	(Δ/σ)_max_ = 0.001
*S* = 1.16	Δρ_max_ = 1.75 e Å^−^^3^
6710 reflections	Δρ_min_ = −1.18 e Å^−^^3^
363 parameters	

### Special details

**Table:**

Geometry. All e.s.d.'s (except the e.s.d. in the dihedral angle between two l.s. planes) are estimated using the full covariance matrix. The cell e.s.d.'s are taken into account individually in the estimation of e.s.d.'s in distances, angles and torsion angles; correlations between e.s.d.'s in cell parameters are only used when they are defined by crystal symmetry. An approximate (isotropic) treatment of cell e.s.d.'s is used for estimating e.s.d.'s involving l.s. planes.

### Fractional atomic coordinates and isotropic or equivalent isotropic displacement parameters (Å^2^)

**Table:**

	*x*	*y*	*z*	*U*_iso_*/*U*_eq_	
C1	0.6328 (4)	0.0332 (4)	0.3430 (2)	0.0177 (8)	
C2	1.0765 (4)	0.7216 (5)	0.1312 (3)	0.0239 (9)	
C3	1.1293 (5)	0.8324 (5)	0.1536 (3)	0.0319 (10)	
H3A	1.0737	0.9245	0.1213	0.048*	
H3B	1.2309	0.8127	0.1374	0.048*	
H3C	1.117	0.8308	0.2163	0.048*	
C4	1.1869 (5)	0.5745 (5)	0.1384 (3)	0.0377 (12)	
H4A	1.1384	0.509	0.1324	0.057*	
H4B	1.2329	0.5453	0.1954	0.057*	
H4C	1.2597	0.5754	0.0922	0.057*	
C11	0.6713 (4)	0.2154 (4)	0.1495 (2)	0.0142 (7)	
C12	0.7244 (4)	0.0790 (4)	0.1287 (2)	0.0181 (8)	
H12	0.7963	0.0068	0.1637	0.022*	
C13	0.6713 (4)	0.0515 (4)	0.0572 (3)	0.0203 (8)	
H13	0.7079	−0.039	0.0439	0.024*	
C14	0.5630 (4)	0.1580 (4)	0.0043 (2)	0.0203 (8)	
H14	0.5281	0.1394	−0.0445	0.024*	
C15	0.5083 (4)	0.2912 (4)	0.0251 (2)	0.0206 (8)	
H15	0.4347	0.3622	−0.0093	0.025*	
C16	0.5624 (4)	0.3204 (4)	0.0974 (2)	0.0170 (8)	
H16	0.525	0.4109	0.1106	0.02*	
C21	0.9422 (4)	0.2162 (4)	0.2069 (2)	0.0165 (8)	
C22	1.0527 (4)	0.1445 (4)	0.2703 (2)	0.0162 (8)	
H22	1.0277	0.1147	0.3292	0.019*	
C23	1.2001 (4)	0.1179 (4)	0.2450 (3)	0.0198 (8)	
H23	1.2726	0.0712	0.2873	0.024*	
C24	1.2381 (4)	0.1601 (4)	0.1585 (3)	0.0205 (8)	
H24	1.3362	0.1417	0.1422	0.025*	
C25	1.1307 (5)	0.2304 (4)	0.0950 (3)	0.0221 (9)	
H25	1.1571	0.2592	0.0363	0.027*	
C26	0.9827 (4)	0.2581 (4)	0.1189 (3)	0.0204 (8)	
H26	0.9112	0.3047	0.076	0.024*	
C31	0.6668 (4)	0.4369 (4)	0.2389 (2)	0.0158 (8)	
C32	0.7292 (4)	0.5387 (4)	0.1976 (2)	0.0171 (8)	
H32	0.8199	0.5111	0.1692	0.021*	
C33	0.6579 (4)	0.6807 (4)	0.1984 (3)	0.0198 (8)	
H33	0.7011	0.7482	0.171	0.024*	
C34	0.5219 (5)	0.7233 (4)	0.2398 (3)	0.0215 (9)	
H34	0.4733	0.8193	0.2395	0.026*	
C35	0.4598 (4)	0.6231 (4)	0.2813 (2)	0.0198 (8)	
H35	0.3687	0.6511	0.3091	0.024*	
C36	0.5321 (4)	0.4806 (4)	0.2818 (2)	0.0186 (8)	
H36	0.4901	0.4131	0.3111	0.022*	
C41	0.8788 (4)	0.3118 (4)	0.5592 (2)	0.0150 (8)	
C42	1.0024 (4)	0.2448 (4)	0.6094 (2)	0.0184 (8)	
H42	1.047	0.1468	0.6171	0.022*	
C43	1.0612 (4)	0.3262 (4)	0.6493 (2)	0.0215 (9)	
H43	1.1461	0.2824	0.6825	0.026*	
C44	0.9926 (4)	0.4700 (4)	0.6387 (2)	0.0206 (8)	
H44	1.0319	0.5229	0.6652	0.025*	
C45	0.8626 (4)	0.5407 (4)	0.5882 (2)	0.0172 (8)	
C46	0.8031 (4)	0.4598 (4)	0.5459 (2)	0.0143 (7)	
C47	0.6743 (4)	0.5309 (4)	0.4946 (2)	0.0165 (8)	
H47	0.6355	0.4796	0.4664	0.02*	
C48	0.6073 (4)	0.6744 (4)	0.4867 (3)	0.0202 (8)	
H48	0.5224	0.7197	0.4537	0.024*	
C49	0.6656 (4)	0.7544 (4)	0.5281 (3)	0.0211 (9)	
H49	0.6189	0.852	0.5221	0.025*	
C50	0.7902 (4)	0.6894 (4)	0.5769 (2)	0.0192 (8)	
H50	0.8281	0.7437	0.6032	0.023*	
N1	0.8228 (3)	0.2270 (3)	0.51666 (19)	0.0141 (6)	
N7	0.7762 (3)	0.1307 (3)	0.5647 (2)	0.0165 (7)	
O1	0.5680 (3)	−0.0262 (3)	0.31778 (19)	0.0293 (7)	
O2	0.8240 (3)	0.2552 (3)	0.42710 (16)	0.0162 (6)	
O3	0.7281 (3)	0.0595 (3)	0.51892 (16)	0.0172 (6)	
O4	0.9494 (3)	0.7466 (3)	0.1110 (2)	0.0331 (7)	
P1	0.75192 (10)	0.24553 (10)	0.24302 (6)	0.0132 (2)	
Rh1	0.72947 (3)	0.13381 (3)	0.381902 (17)	0.01389 (12)	

### Atomic displacement parameters (Å^2^)

**Table:**

	*U*^11^	*U*^22^	*U*^33^	*U*^12^	*U*^13^	*U*^23^
C1	0.0155 (19)	0.015 (2)	0.0185 (19)	−0.0022 (16)	0.0009 (15)	0.0012 (15)
C2	0.019 (2)	0.031 (2)	0.019 (2)	−0.0048 (18)	0.0013 (16)	−0.0045 (17)
C3	0.028 (2)	0.032 (3)	0.034 (2)	−0.010 (2)	0.0047 (19)	−0.004 (2)
C4	0.028 (3)	0.029 (3)	0.056 (3)	−0.001 (2)	−0.007 (2)	−0.018 (2)
C11	0.0118 (17)	0.017 (2)	0.0139 (17)	−0.0053 (15)	0.0017 (14)	−0.0026 (14)
C12	0.0152 (19)	0.016 (2)	0.0204 (19)	−0.0018 (16)	−0.0023 (15)	−0.0032 (15)
C13	0.024 (2)	0.017 (2)	0.021 (2)	−0.0059 (17)	0.0032 (16)	−0.0079 (16)
C14	0.022 (2)	0.027 (2)	0.0160 (18)	−0.0123 (18)	−0.0006 (15)	−0.0065 (16)
C15	0.019 (2)	0.025 (2)	0.0168 (19)	−0.0090 (17)	−0.0019 (15)	0.0012 (16)
C16	0.0164 (19)	0.015 (2)	0.0202 (19)	−0.0058 (16)	−0.0027 (15)	−0.0035 (15)
C21	0.0148 (18)	0.0135 (19)	0.0211 (19)	−0.0032 (15)	−0.0008 (15)	−0.0055 (15)
C22	0.0146 (19)	0.015 (2)	0.0178 (18)	−0.0037 (15)	0.0039 (14)	−0.0038 (15)
C23	0.018 (2)	0.021 (2)	0.022 (2)	−0.0057 (16)	0.0004 (16)	−0.0064 (16)
C24	0.0154 (19)	0.017 (2)	0.030 (2)	−0.0062 (16)	0.0063 (16)	−0.0098 (17)
C25	0.026 (2)	0.024 (2)	0.018 (2)	−0.0099 (18)	0.0074 (16)	−0.0076 (17)
C26	0.022 (2)	0.020 (2)	0.021 (2)	−0.0073 (17)	−0.0044 (16)	−0.0044 (16)
C31	0.0189 (19)	0.0141 (19)	0.0121 (17)	−0.0022 (15)	−0.0062 (14)	−0.0010 (14)
C32	0.019 (2)	0.018 (2)	0.0162 (18)	−0.0081 (16)	−0.0061 (15)	−0.0025 (15)
C33	0.025 (2)	0.015 (2)	0.022 (2)	−0.0097 (17)	−0.0054 (16)	−0.0024 (16)
C34	0.027 (2)	0.012 (2)	0.024 (2)	−0.0001 (16)	−0.0081 (17)	−0.0067 (16)
C35	0.017 (2)	0.017 (2)	0.0195 (19)	0.0021 (16)	−0.0019 (15)	−0.0037 (16)
C36	0.022 (2)	0.018 (2)	0.0151 (18)	−0.0068 (17)	0.0020 (15)	−0.0020 (15)
C41	0.0142 (18)	0.019 (2)	0.0144 (18)	−0.0070 (16)	0.0014 (14)	−0.0077 (15)
C42	0.018 (2)	0.017 (2)	0.0162 (18)	−0.0006 (16)	0.0003 (15)	−0.0040 (15)
C43	0.019 (2)	0.027 (2)	0.0180 (19)	−0.0052 (17)	−0.0008 (15)	−0.0054 (16)
C44	0.022 (2)	0.026 (2)	0.0190 (19)	−0.0122 (18)	−0.0008 (16)	−0.0068 (16)
C45	0.0175 (19)	0.020 (2)	0.0180 (19)	−0.0092 (16)	0.0047 (15)	−0.0090 (16)
C46	0.0123 (18)	0.0146 (19)	0.0169 (18)	−0.0047 (15)	0.0023 (14)	−0.0057 (15)
C47	0.0158 (19)	0.016 (2)	0.0189 (19)	−0.0059 (16)	−0.0003 (15)	−0.0041 (15)
C48	0.0142 (19)	0.019 (2)	0.023 (2)	−0.0009 (16)	0.0011 (15)	−0.0044 (16)
C49	0.019 (2)	0.016 (2)	0.026 (2)	−0.0036 (16)	0.0058 (16)	−0.0044 (16)
C50	0.027 (2)	0.020 (2)	0.0180 (19)	−0.0153 (18)	0.0090 (16)	−0.0086 (16)
N1	0.0145 (16)	0.0126 (16)	0.0143 (15)	−0.0028 (13)	0.0005 (12)	−0.0036 (12)
N7	0.0158 (16)	0.0121 (16)	0.0190 (16)	−0.0019 (13)	−0.0038 (13)	−0.0009 (13)
O1	0.0324 (18)	0.0344 (19)	0.0309 (16)	−0.0209 (15)	−0.0054 (13)	−0.0089 (14)
O2	0.0209 (14)	0.0182 (14)	0.0101 (12)	−0.0076 (12)	0.0009 (10)	−0.0023 (10)
O3	0.0206 (14)	0.0158 (14)	0.0130 (13)	−0.0035 (11)	−0.0021 (10)	−0.0015 (10)
O4	0.0233 (17)	0.040 (2)	0.0330 (17)	−0.0042 (14)	−0.0052 (13)	−0.0079 (14)
P1	0.0131 (5)	0.0114 (5)	0.0141 (5)	−0.0018 (4)	−0.0013 (4)	−0.0035 (4)
Rh1	0.01397 (18)	0.01172 (19)	0.01518 (18)	−0.00255 (13)	−0.00048 (12)	−0.00361 (12)

### Geometric parameters (Å, °)

**Table:**

C1—O1	1.146 (5)	C31—C36	1.389 (5)
C1—Rh1	1.817 (4)	C31—P1	1.832 (4)
C2—O4	1.226 (5)	C32—C33	1.379 (5)
C2—C3	1.497 (6)	C32—H32	0.93
C2—C4	1.507 (6)	C33—C34	1.388 (6)
C3—H3A	0.96	C33—H33	0.93
C3—H3B	0.96	C34—C35	1.371 (6)
C3—H3C	0.96	C34—H34	0.93
C4—H4A	0.96	C35—C36	1.381 (5)
C4—H4B	0.96	C35—H35	0.93
C4—H4C	0.96	C36—H36	0.93
C11—C16	1.385 (5)	C41—C42	1.372 (5)
C11—C12	1.407 (5)	C41—C46	1.414 (5)
C11—P1	1.823 (4)	C41—N1	1.448 (5)
C12—C13	1.373 (5)	C42—C43	1.410 (6)
C12—H12	0.93	C42—H42	0.93
C13—C14	1.393 (5)	C43—C44	1.368 (6)
C13—H13	0.93	C43—H43	0.93
C14—C15	1.377 (6)	C44—C45	1.424 (5)
C14—H14	0.93	C44—H44	0.93
C15—C16	1.398 (5)	C45—C50	1.416 (5)
C15—H15	0.93	C45—C46	1.433 (5)
C16—H16	0.93	C46—C47	1.423 (5)
C21—C26	1.394 (5)	C47—C48	1.367 (5)
C21—C22	1.407 (5)	C47—H47	0.93
C21—P1	1.825 (4)	C48—C49	1.410 (6)
C22—C23	1.401 (5)	C48—H48	0.93
C22—H22	0.93	C49—C50	1.367 (6)
C23—C24	1.367 (5)	C49—H49	0.93
C23—H23	0.93	C50—H50	0.93
C24—C25	1.387 (6)	N1—N7	1.281 (4)
C24—H24	0.93	N1—O2	1.346 (4)
C25—C26	1.400 (6)	N7—O3	1.323 (4)
C25—H25	0.93	O2—Rh1	2.026 (3)
C26—H26	0.93	O3—Rh1	2.082 (2)
C31—C32	1.385 (5)	P1—Rh1	2.2240 (11)
			
O1—C1—Rh1	177.7 (4)	C32—C33—H33	119.8
O4—C2—C3	122.5 (4)	C34—C33—H33	119.8
O4—C2—C4	121.1 (4)	C35—C34—C33	119.6 (4)
C3—C2—C4	116.3 (4)	C35—C34—H34	120.2
C2—C3—H3A	109.5	C33—C34—H34	120.2
C2—C3—H3B	109.5	C34—C35—C36	120.2 (4)
H3A—C3—H3B	109.5	C34—C35—H35	119.9
C2—C3—H3C	109.5	C36—C35—H35	119.9
H3A—C3—H3C	109.5	C35—C36—C31	120.7 (4)
H3B—C3—H3C	109.5	C35—C36—H36	119.6
C2—C4—H4A	109.5	C31—C36—H36	119.6
C2—C4—H4B	109.5	C42—C41—C46	123.7 (3)
H4A—C4—H4B	109.5	C42—C41—N1	118.5 (3)
C2—C4—H4C	109.5	C46—C41—N1	117.8 (3)
H4A—C4—H4C	109.5	C41—C42—C43	119.3 (4)
H4B—C4—H4C	109.5	C41—C42—H42	120.3
C16—C11—C12	118.5 (3)	C43—C42—H42	120.3
C16—C11—P1	123.3 (3)	C44—C43—C42	119.5 (4)
C12—C11—P1	118.1 (3)	C44—C43—H43	120.2
C13—C12—C11	120.7 (4)	C42—C43—H43	120.2
C13—C12—H12	119.7	C43—C44—C45	121.9 (4)
C11—C12—H12	119.7	C43—C44—H44	119.1
C12—C13—C14	120.5 (4)	C45—C44—H44	119.1
C12—C13—H13	119.7	C50—C45—C44	122.5 (3)
C14—C13—H13	119.7	C50—C45—C46	118.3 (3)
C15—C14—C13	119.3 (3)	C44—C45—C46	119.2 (3)
C15—C14—H14	120.3	C41—C46—C47	124.5 (3)
C13—C14—H14	120.3	C41—C46—C45	116.3 (3)
C14—C15—C16	120.5 (4)	C47—C46—C45	119.2 (3)
C14—C15—H15	119.7	C48—C47—C46	120.3 (4)
C16—C15—H15	119.7	C48—C47—H47	119.8
C11—C16—C15	120.4 (4)	C46—C47—H47	119.8
C11—C16—H16	119.8	C47—C48—C49	120.7 (4)
C15—C16—H16	119.8	C47—C48—H48	119.6
C26—C21—C22	118.7 (3)	C49—C48—H48	119.6
C26—C21—P1	122.8 (3)	C50—C49—C48	120.3 (4)
C22—C21—P1	118.4 (3)	C50—C49—H49	119.8
C23—C22—C21	120.2 (3)	C48—C49—H49	119.8
C23—C22—H22	119.9	C49—C50—C45	121.2 (4)
C21—C22—H22	119.9	C49—C50—H50	119.4
C24—C23—C22	120.4 (4)	C45—C50—H50	119.4
C24—C23—H23	119.8	N7—N1—O2	123.9 (3)
C22—C23—H23	119.8	N7—N1—C41	119.5 (3)
C23—C24—C25	120.2 (4)	O2—N1—C41	116.7 (3)
C23—C24—H24	119.9	N1—N7—O3	114.3 (3)
C25—C24—H24	119.9	N1—O2—Rh1	110.0 (2)
C24—C25—C26	120.3 (4)	N7—O3—Rh1	113.7 (2)
C24—C25—H25	119.8	C11—P1—C21	102.64 (17)
C26—C25—H25	119.8	C11—P1—C31	103.59 (16)
C21—C26—C25	120.2 (4)	C21—P1—C31	106.98 (17)
C21—C26—H26	119.9	C11—P1—Rh1	121.83 (13)
C25—C26—H26	119.9	C21—P1—Rh1	113.03 (12)
C32—C31—C36	118.7 (4)	C31—P1—Rh1	107.63 (12)
C32—C31—P1	124.3 (3)	C1—Rh1—O2	176.15 (13)
C36—C31—P1	117.0 (3)	C1—Rh1—O3	101.74 (14)
C33—C32—C31	120.4 (4)	O2—Rh1—O3	77.74 (10)
C33—C32—H32	119.8	C1—Rh1—P1	90.54 (12)
C31—C32—H32	119.8	O2—Rh1—P1	89.92 (8)
C32—C33—C34	120.3 (4)	O3—Rh1—P1	167.66 (8)
